# Clinical safety and feasibility of a novel implantable neuroimmune modulation device for the treatment of rheumatoid arthritis: initial results from the randomized, double-blind, sham-controlled RESET-RA study

**DOI:** 10.1186/s42234-023-00138-x

**Published:** 2024-03-13

**Authors:** Daniel Peterson, Mark Van Poppel, Warren Boling, Perry Santos, Jason Schwalb, Howard Eisenberg, Ashesh Mehta, Heather Spader, James Botros, Frank D. Vrionis, Andrew Ko, P. David Adelson, Bradley Lega, Peter Konrad, Guillermo Calle, Fernando L. Vale, Richard Bucholz, R. Mark Richardson

**Affiliations:** 1 Neurosurgery, Austin Neurosurgeons (Arise Medical Center), Austin, TX USA; 2 Neurosurgery, Carolina Neurosurgery & Spine Associates, Charlotte, NC USA; 3 Neurosurgery, Loma Linda University Health, Loma Linda, CA USA; 4 Integris Health Baptist Medical Center, Head and Neck Surgery, Oklahoma City, OK USA; 5 Neurosurgery, Henry Ford Medical Group, Detroit, MI USA; 6 Neurosurgery, University of Maryland Medical Center, Baltimore, MD USA; 7 The Feinstein Institutes for Medical Research, Neurosurgery, Donald and Barbara Zucker School of Medicine at Hofstra/Northwell, Manhasset, NY USA; 8 Neurosurgery, University of Virginia, Charlottesville, VA USA; 9 Neurosurgery, Marcus Neuroscience Institute, Boca Raton, FL USA; 10 Neurosurgery, University of Washington, Seattle, WA USA; 11 Neurosurgery, Phoenix Children’s Hospital, Phoenix, AZ USA; 12 Neurological Surgery, The University of Texas Southwestern Medical Center, Dallas, TX USA; 13 Rockefeller Neuroscience Institute, Neurosurgery, West Virginia University Medicine, Morgantown, WV USA; 14 Product Development, SetPoint Medical, Valencia, CA USA; 15 Medical College of Georgia at Augusta University, Augusta, GA USA; 16 Division of Neurological Surgery, St. Louis University, St. Louis, MO USA; 17 Neurosurgery, Massachusetts General Hospital, Boston, MA USA; 18 Neurosurgery, University of New Mexico, Albuquerque, NM USA

**Keywords:** Vagus nerve stimulation, Rheumatoid arthritis, Inflammatory reflex, Neuroimmune modulation

## Abstract

**Background:**

Rheumatoid arthritis (RA) is a chronic inflammatory autoimmune disease that causes persistent synovitis, bone damage, and progressive joint destruction. Neuroimmune modulation through electrical stimulation of the vagus nerve activates the inflammatory reflex and has been shown to inhibit the production and release of inflammatory cytokines and decrease clinical signs and symptoms in RA. The RESET-RA study was designed to determine the safety and efficacy of an active implantable device for treating RA.

**Methods:**

The RESET-RA study is a randomized, double-blind, sham-controlled, multi-center, two-stage pivotal trial that enrolled patients with moderate-to-severe RA who were incomplete responders or intolerant to at least one biologic or targeted synthetic disease-modifying anti-rheumatic drug. A neuroimmune modulation device (SetPoint Medical, Valencia, CA) was implanted on the left cervical vagus nerve within the carotid sheath in all patients. Following post-surgical clearance, patients were randomly assigned (1:1) to active stimulation or non-active (control) stimulation for 1 min once per day. A predefined blinded interim analysis was performed in patients enrolled in the study’s initial stage (Stage 1) that included demographics, enrollment rates, device implantation rates, and safety of the surgical procedure, device, and stimulation over 12 weeks of treatment.

**Results:**

Sixty patients were implanted during Stage 1 of the study. All device implant procedures were completed without intraoperative complications, infections, or surgical revisions. No unanticipated adverse events were reported during the perioperative period and at the end of 12 weeks of follow-up. No study discontinuations were due to adverse events, and no serious adverse events were related to the device or stimulation. Two serious adverse events were related to the implantation procedure: vocal cord paresis and prolonged hoarseness. These were reported in two patients and are known complications of surgical implantation procedures with vagus nerve stimulation devices. The adverse event of vocal cord paresis resolved after vocal cord augmentation injections with filler and speech therapy. The prolonged hoarseness had improved with speech therapy, but mild hoarseness persists.

**Conclusions:**

The surgical procedures for implantation of the novel neuroimmune modulation device for the treatment of RA were safe, and the device and its use were well tolerated.

**Trial registration:**

NCT04539964; August 31, 2020.

**Supplementary Information:**

The online version contains supplementary material available at 10.1186/s42234-023-00138-x.

## Background

Rheumatoid arthritis (RA) is a chronic, systemic inflammatory autoimmune disease that affects about 1% of the adult population worldwide, with up to a three-fold higher prevalence in women and a typical onset between the ages of 30 and 50 years (Smolen 2020; Smolen et al. 2016). Chronic inflammation of the joints is a hallmark of the disease, occurring in the synovial tissue and manifesting as swelling, pain, stiffness, and restricted mobility. This inflammation often destroys articular cartilage and juxta-articular bone, causing irreversible joint damage (Schett and Gravallese 2012). Despite the availability of multiple classes of disease-modifying anti-rheumatic drugs (DMARDs) for treatment of RA, long term persistence on therapy is poor with up to 50% of patients discontinuing their therapy after 2 years due to lack of efficacy, toxicity and poor compliance (Ebina et al. 2019). The annual economic cost of RA to both the individual and society is significant. In the US, the estimated annual direct health care costs for RA resulted in total incremental costs of $22.3 billion (2008 USD) (Kawatkar et al. 2012). Drug costs comprised the main component (up to 87%) (Hsieh et al. 2020) of the direct costs of RA care, with multiple drug-refractory disease significantly contributing to this high economic burden (Strand et al. 2018). An urgent need exists to develop safer, compliance-friendly and cost-effective, differentiated RA therapies and expand treatment approaches for non-responders to first-line DMARDs.

The coordination and control of the autonomic nervous system over peripheral inflammation has been identified through a recent convergence in immunology, physiology, and neuroscience (Tracey 2009). This central control is mediated through nerves with interoceptive functions, such as the vagus nerve, which sense peripheral inflammation, followed by reflexive activation of the cholinergic anti-inflammatory pathway – motor signaling through the efferent vagus nerve that affect innate immune function. These signals are transmitted across the celiac plexus, through the splenic nerve and to the spleen, directly affecting neurotransmitter-sensitive immunocytes (Tracey 2009). The paired sensory and cholinergic anti-inflammatory pathways are together termed the inflammatory reflex and inhibit the production of TNF, IL-1, IL-6, and other proinflammatory cytokines in immune cells within the reticuloendothelial system, attenuating peripheral inflammation (Tracey 2002). Evidence implicating autonomic imbalance as a key underlying characteristic of several inflammatory diseases has led to the clinical development of electrical stimulation of the vagus nerve as a potential therapeutic option for autoimmune conditions such as RA (Koopman et al. 2017) and Crohn's disease (Bonaz et al. 2016; D'Haens et al. 2023). Neuroimmune modulation by intermittent stimulation of the left cervical vagus nerve has been shown to inhibit the production of inflammatory cytokines and decrease signs and symptoms in both preclinical disease models (Borovikova et al. 2000; Pavlov et al. 2018) and in small prospective clinical studies of RA and Crohn’s disease (Koopman et al. 2016; Bonaz et al. 2016; Genovese et al. 2020; D'Haens et al. 2023).

A novel neuroimmune modulation system (SetPoint Medical, Valencia, CA) explicitly designed to activate the inflammatory reflex for the treatment of RA was developed and tested in a first-in-human clinical study (Genovese et al. 2020). Results indicated that the device was safe and well tolerated. Furthermore, the treatment led 50% of patients with multiple drug-refractory RA to achieve clinically meaningful responses in disease activity and reductions in inflammatory cytokines. Based on these findings, the RESET-RA study (NCT04539964) was designed to further evaluate this approach and device in a larger sham-controlled study. This report describes the safety of surgical implantation, treatment, and the novel device in the first 60 patients enrolled in the RESET-RA study through the primary endpoint (Week 12 Visit). Clinical efficacy and patient-reported effectiveness outcomes will be presented in a forthcoming report.

## Methods

### Study design and conduct

**Fig. 1:**
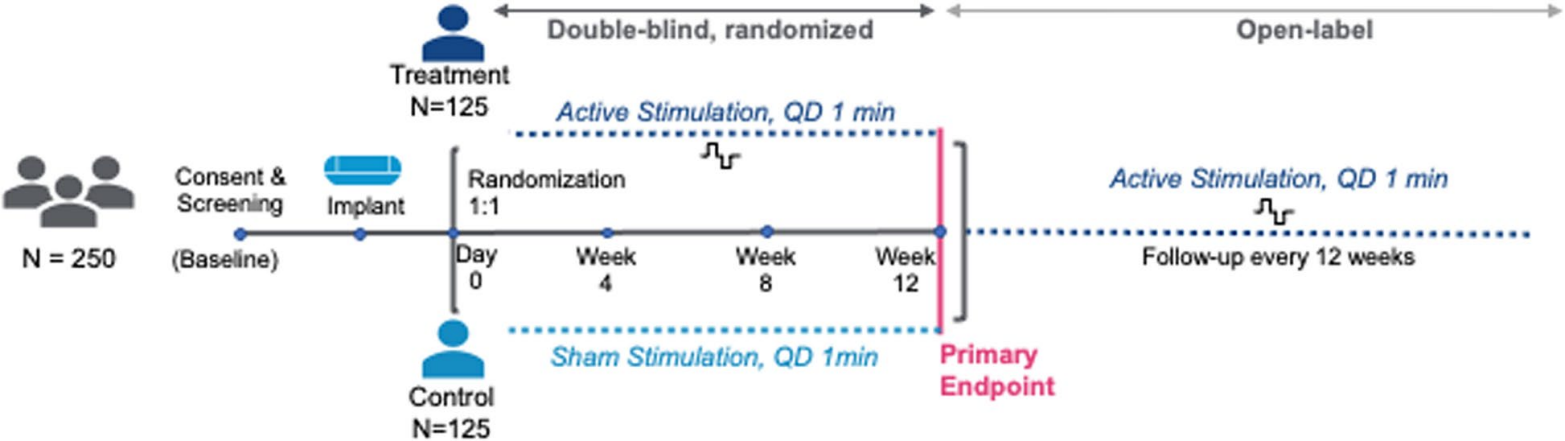
RESET-RA study design

The RESET-RA study is a randomized, double-blind, sham-controlled, multi-center, two-stage study aimed at evaluating the safety and efficacy of a novel neuroimmune modulatory treatment in patients with moderate to severe RA who have experienced incomplete response or intolerance to one or more biologic or targeted synthetic DMARDs. The efficacy primary endpoint is after 12 weeks of stimulation, and patients will then be followed across a long-term extension period (180 weeks). The study plans to enroll up to 250 patients across two consecutive stages at up to 45 US clinical study sites (Fig. 1). Stage 1, comprising the first 60 patients enrolled, was initiated in January 2021 (first patient implanted) and completed enrollment in March 2022. A preplanned interim analysis of Stage 1 was conducted to check for safety risks and a lack of efficacy before enrolling patients in Stage 2. Additionally, a stopping rule, predefined as a difference of less than 10% between the treatment and sham groups in the proportion of American College of Rheumatology 20 (ACR20) responders at the Week 12 primary endpoint, was established to prevent the continuation of a trial with a projected low probability of success. The stopping rule was not met, and the US Food and Drug Administration approved the initiation of Stage 2 of the study in July 2022.

The study followed the Declaration of Helsinki and the International Council for Harmonization Good Clinical Practice guidelines. The institutional review board or ethics committee approved the study protocol at each study site. All subjects provided written informed consent before participating in any study activities.

### Patient characteristics

Study candidates were recruited from the investigators’ clinical practices and through referrals and advertising and gave their full written informed consent before any study activities. Eligible patients were 22 to 75 years old at the time of consent with a diagnosis of active moderate-to-severe RA defined as at least 4/28 tender joints and 4/28 swollen joints and an inadequate response or intolerance to at least one biologic or targeted synthetic DMARD (e.g., an anti-TNF antibody or JAK inhibitor). Patients were required to receive stable treatment with at least one conventional synthetic DMARD, e.g., methotrexate, for at least 12 weeks before consent. Patients with implanted active medical devices (e.g., cardiac pacemakers, automatic implantable cardioverter-defibrillators, other neurostimulators) or likely need for implantation of such devices within 6 months were excluded from the study. Full inclusion and exclusion criteria and restricted medications and procedures are detailed in the Additional file 1.

### Device and treatment

**Fig. 2:**
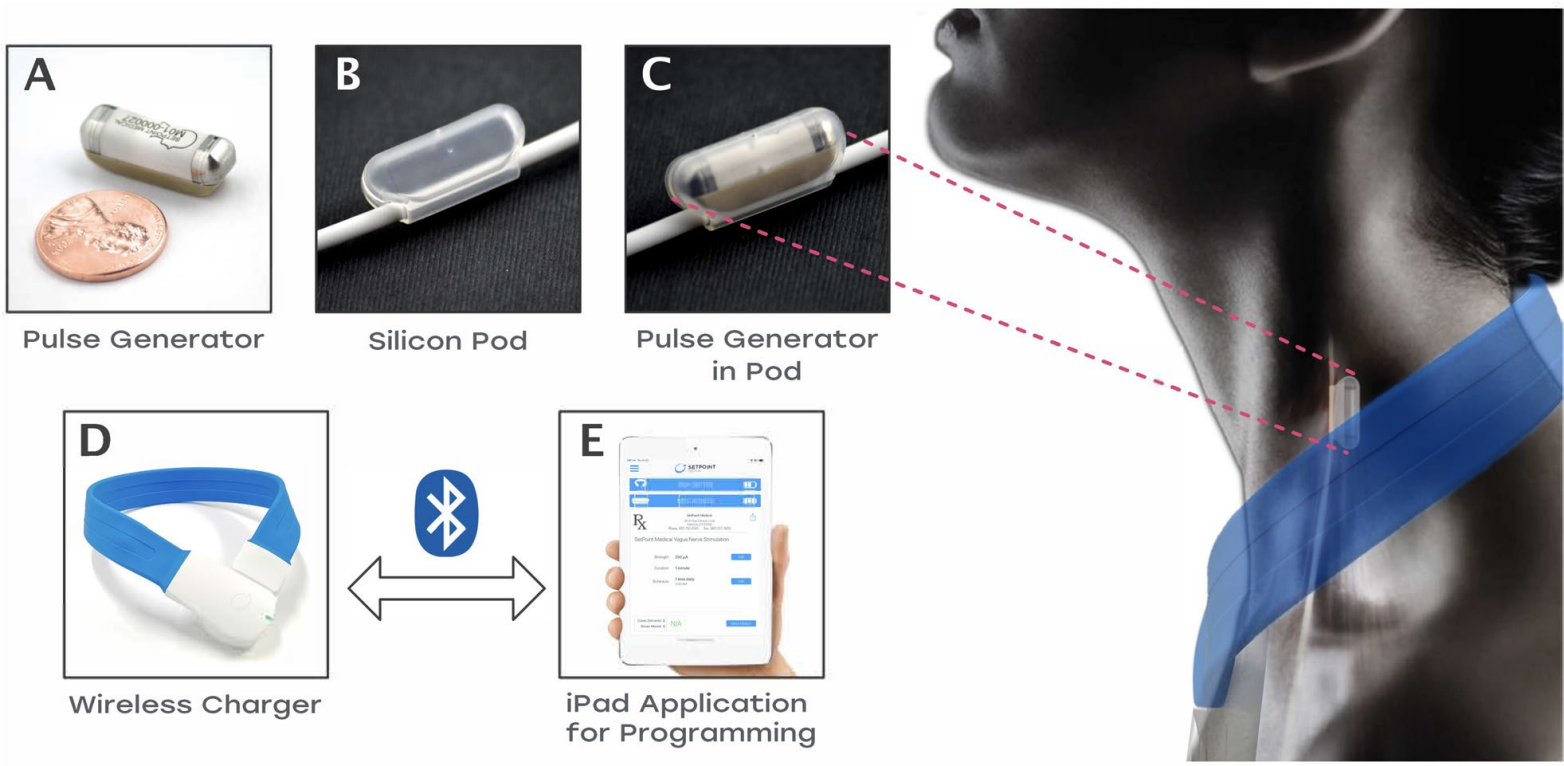
Neuroimmune modulation system

The neuroimmune modulation system (SetPoint Medical, Valencia, CA) consists of 2 implanted components: a miniaturized pulse generator with integrated electrodes and a silicon pod that positions the pulse generator on the left vagus nerve; and two external components: the wireless charger and an iPad application for programming the pulse generator (Fig. 2). The pulse generator is implanted on the left cervical vagus nerve within the carotid sheath. The silicon pod holds the pulse generator in close apposition to the nerve and electrically insulates the device from surrounding tissues. Once implanted, the vagus nerve fits into a groove on the base of the pulse generator, where the electrodes are oriented in direct apposition to the nerve for efficient stimulation. The device is powered by a rechargeable lithium-ion battery with a usage life of at least 10 years.

The pulse generator is recharged using a proprietary radio frequency-based external wireless charger worn around the neck for a few minutes weekly. The charger also provides wireless telemetry for the transmission and receipt of information with an Apple® iPad-based software application, which healthcare professionals use to program and monitor implants.

The stimulation parameters (10 Hz pulse frequency, 0.25 ms pulse width, 60-second pulse train duration, once/day) were based on extensive preclinical work in animal models of inflammation (Levine et al. 2020) and were specifically designed to activate the inflammatory reflex to decrease systemic inflammation. Patient-specific pulse amplitude was programmed to be delivered automatically based on the titration scheme described below.

Following pre-surgical clearance, all patients were implanted in an outpatient setting and returned to the surgeon 14–21 days later for a post-surgical checkup and clearance. Following post-surgical clearance, patients were randomly assigned (1:1) to active stimulation or non-active (control) stimulation for 1 min once per day. Non-active stimulation devices operated identical to the active stimulation devices but delivered sham stimulation at 0 V to maintain study blinding. After completing Week 12 assessments (typical length of a therapeutics trial in rheumatoid arthritis), the long-term extension period of the study commenced, control group patients crossed over to active stimulation, and all patients received active stimulation for 1 min once per day. Treatment assignment was stratified by prior Janus kinase inhibitor (JAKi) treatment and joint count following the implant procedure. Randomization was implemented centrally through an interactive response technology. Patients, investigators, care providers, study staff, and sponsor (outside of select remote programmers blinded to individual patient outcomes) were blinded to treatment allocation.

### Surgical implantation procedure

The surgery to place the neuroimmune modulation device on the left vagus nerve is performed in an outpatient procedure with an approximate duration of 60 to 90 min. The implantation site was a 3 cm segment of the left vagus nerve approximately halfway between the clavicle and the mastoid process. Optimal positioning of the pulse generator was identified preoperatively by placing the wireless charger on the patient’s neck, marking the upper and lower boundaries of the charger, and noting the markings in reference to anatomical landmarks. These markings ensured proper coupling between the charger and the implanted pulse generator for charging and programming. Video 1 demonstrates the surgical implantation procedure. First, a transverse incision was made within a skin crease on the left side of the neck. Next, the fascia and musculature anterior to and around the sternocleidomastoid’s medial border were dissected to expose the carotid sheath.

The left vagus nerve was then identified to allow the insertion of the pulse generator and the silicon pod. The ideal location for implantation is a nerve segment clear of branches below the separation of the superior and inferior cervical cardiac branches at approximately the C4-C6 cervical vertebrae level. The tissue surrounding the carotid sheath was dissected circumferentially to isolate the nerve while minimizing tension on the nerve. Lateral dissection was performed until at least 3 cm of the nerve was exposed.

The silicon pod was inserted underneath the nerve with forceps or hemostats and then released and positioned around the nerve. The pulse generator was inserted into the pod. The pod was closed and checked to ensure the nerve was correctly seated under the pulse generator and positioned through the proximal and distal openings of the pod without entrapment of nerve branches or surrounding tissue. The pod was secured through pre-existing suture holes using a non-absorbable 5 − 0 prolene suture on a non-cutting needle. Finally, the pocket was flushed with an antibiotic solution, and the fascia, musculature, and skin were closed.

### Device programming

Following the surgical postoperative check two to three weeks following recovery from the implant procedure, patients reported to the study’s rheumatology site to be randomized to a treatment assignment and have their stimulation programmed. A study staff member at the rheumatology site programmed the stimulation.

Patients wore the wireless charger around their neck during programming to enable telemetry with their pulse generator through the charger and the iPad-based programming app. Once the app was connected to the pulse generator, an analysis was performed to assess current prescription settings, the total number of doses delivered, the number of doses missed, the battery level of the implant, and the impedance level.

Device programming was performed at the Day 0 (randomization), Week 1, Week 2, and Week 3 visits. The programming was an iterative process during which short test stimulations were administered, and the patient’s tolerability was evaluated. Next, the stimulation output current (amplitude) was incremented and reassessed for tolerability. This was repeated to the upper comfort level or to the maximum allowable current level specified for the visit, whichever came first. The maximum allowable current for the four successive titration visits were 850, 1450, 2050, and 2500 µA. No stimulation pulse parameters were modified other than the pulse amplitude to the patient’s upper comfort level. Once the optimal setting was determined at each visit, the patient’s device parameters were set to deliver the assigned treatment stimulations for one minute once per day.

To maintain study blinding, the chargers were designed to provide visual (LED light) and auditory (beeping) signals for all patients during programming. Patients were informed that they may or may not feel any sensations during programming and stimulation and were blinded to when their stimulation would be delivered.

### Safety data

Reported here is safety information related to the neuroimmune modulation system, treatment (combined active and sham), and the surgical implantation procedure from the time of consent through the Week 12 visit. Adverse events (AEs) were evaluated and categorized in terms of their seriousness, severity, causality (i.e., relationship to the implant procedure, device, and stimulation), and whether they were anticipated. AEs were coded according to the Medical Dictionary for Regulatory Activities, version 21.0. Clinical effectiveness outcomes will be presented in a subsequent report upon the completion of Stage 2 of the study. Study data were recorded and stored in compliance with local regulations and were periodically monitored by the study sponsor for quality and completeness. Procedures were employed to prevent compromise of patient confidentiality. Descriptive statistics were calculated and expressed as mean ± standard deviation.

## Results

### Study participants

**Fig. 3:**
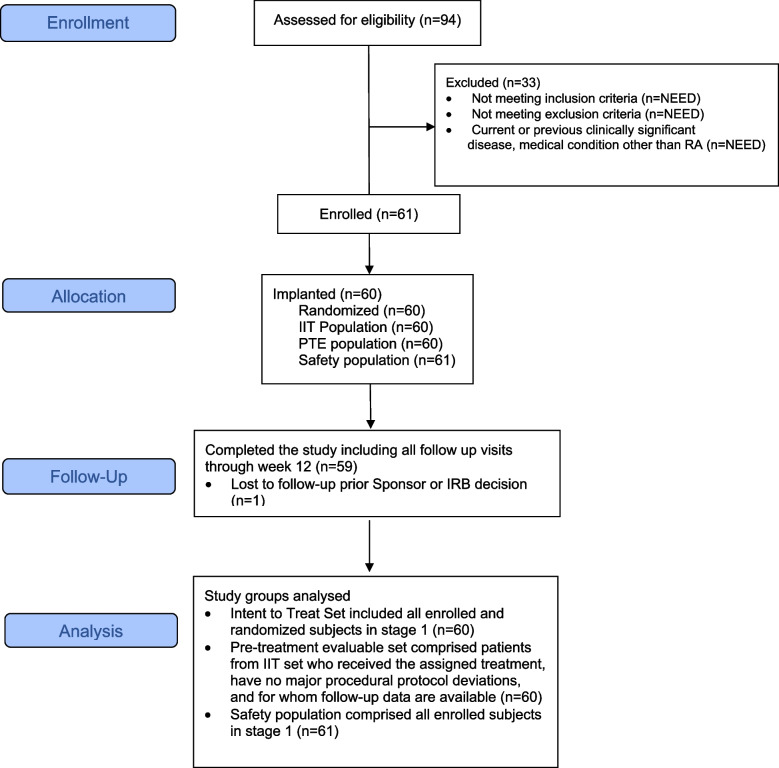
CONSORT flow diagram

During the study, 94 patients were screened, 61 enrolled and 60 were implanted and randomized in Stage 1. One patient enrolled in the study but discontinued prior to implantation due to the investigator’s decision. Overall, 98% of patients implanted completed 12 weeks of follow-up, with one (1) patient missing their Week 12 visit due to an unrelated adverse event. Figure 3 summarizes the patient CONSORT flow diagram. Most patients were white (88.3%) and female (85.0%), with a median age of 57.9 years and a mean rheumatoid arthritis disease duration of 14.6 years (Table 1). All patients enrolled had at least one prior biologic DMARD or JAKi at baseline, with 45% treated with one prior biologic DMARD or JAKi. Mean Disease Activity Score 28-C-reactive protein (DAS28-CRP) and Clinical Disease Activity Index (CDAI) were 5.3 and 37.9, respectively, indicating patients had high RA disease activity at study entry.

**Table 1:** Baseline demographics and disease characteristics^ab^

	ALL *n* = 60
Sex at birth, n (%), female	51 (85.0)
Age, years	57.9 ± 9.25
Body mass index, kg/m^2^	31.0 ± 6.13
Race, n (%)	
White	53 (88.3)
Asian	2 (3.3)
American Indian/Alaska Native	0 (0)
Black/African American	4 (6.7)
Other	1 (1.7)
Ethnicity, n (%)	
Not Hispanic or Latino	49 (81.7)
Duration of RA diagnosis, years	14.6 ± 10.78
hsCRP, mg/L	7.93 ± 11.98
RF positive, n (%)	29 (48.3)
ACPA positive, n (%)	32 (53.3)
Number of prior b/ts DMARDs	2.5 ± 1.96
Number of prior b/ts DMARDs	
1	27 (45.0%)
2	10 (16.7%)
≥3	23 (38%)
Prior b/ts DMARDs	
Anti-IL-1 agents	2 (3.3%)
Anti-IL-6 agents	13 (21.7%)
Anti-TNF agents	55 (91.7%)
B-cell depleting agents	7 (11.7%)
JAKi	8 (13.3%)
CTLA4-Ig	18 (30.0%)
DAS28(CRP)	5.3 ± 0.88
CDAI	37.9 ± 12.24
SJC28	11.0 ± 5.57
TJC28	14.6 ± 6.99
HAQ-DI	1.4 ± 0.55

^a^Plus-minus values are means ± SD

^b^Calculated based on ITT population

### Safety

All implant procedures were completed without intraoperative complications, infections, or surgical revisions. No unanticipated adverse device effects were reported. During the first 12 weeks of the study, adverse events (AEs) occurred in 40/61 (65.5%) patients, with 121 total AEs reported. Most AEs were nonserious (*N* = 114), mild (*N* = 62), or moderate (*N* = 50) severity and unrelated (*N* = 101) to the investigational device and procedure (Table 2). Frequently reported AEs (occurring in ≥ 5% of patients) consisted of COVID-19 (11.5%), and worsening of RA/RA flare (11.5%). No study discontinuations were due to AEs, and no patients died during the study. Unrelated serious AE (SAE) (*N* = 5) included; acute osteomyelitis, thoracic vertebral fracture, sternal fracture, gastrointestinal hemorrhage, and cervical spinal stenosis. No SAEs were directly related to the device or stimulation.

**Table 2:** Adverse events and serious adverse events by type and relations to study device through Week 12 (safety population)

AE Category MeDRA (v.23.1) preferred term	Number of events (% of 61 enrolled subjects) n (%)
**Any AE**	40 (65.6)
**Unrelated AE**	38 (62.3)
**Related AE**	11 (18)
**Implant Procedure Related AE**	12 (14.8)
Implant site hypoaesthesia	1 (1.6)
Implant site inflammation	1 (1.6)
Swelling	1 (1.6)
Incision site swelling	1 (1.6)
Suture related complication	1 (1.6)
Hypoaesthesia	1 (1.6)
Paraesthesia	1 (1.6)
Vocal cord paresis	1 (1.6)
Dysphonia (Hoarseness)	1 (1.6)
Oropharyngeal pain	1 (1.6)
Rash	1 (1.6)
Scar pain	1 (1.6)
**Stimulation Related AE**	3 (4.9)
Medical device pain	1 (1.6)
Dysphonia (Hoarseness)	1 (1.6)
Dermatitis contact	1 (1.6)
**Any Unrelated, SAE**	5 (8.2)
Acute Osteomyelitis	1 (1.6)
Thoracic vertebral fracture	1 (1.6)
Sternal fracture	1 (1.6)
Gastrointestinal hemorrhage	1 (1.6)
Cervical spinal stenosis	1 (1.6)
**Implant Procedure Related SAE**	2 (3.3)
Vocal cord paresis	1 (1.6)
Dysphonia (Hoarseness)	1 (1.6)
**Device Related SAE**	0
**Stimulation Related SAE**	0
**AEs Leading to study discontinuation**	0
**Deaths**	0

Given in the table are subject counts and percentages. At each level of summation, subjects are counted only once

AEs related to the implant procedure, device, and stimulation were generally mild. Two (2) SAEs related to the implant procedure were reported in two patients: vocal cord paresis and hoarseness. Both are known complications of surgical implantation procedures with FDA-approved vagus nerve stimulation devices. Vocal cord paresis was reported post-operatively in one patient, associated with swelling at the implantation site and hoarseness eight days after the implant procedure. The patient was prescribed prednisone, and the swelling resolved 13 days post-implant. An otolaryngologic evaluation occurred 28 days after the implant procedure and left vocal cord paresis was documented. Treatment included injectable hyaluronic acid filler to temporarily medialize the vocal cord, along with speech therapy. The patient received a total of three injections over three months. The event resolved ten months following onset.

The patient with hoarseness reported a persistent raspy voice following the implant procedure that remained unresolved. A laryngoscopy was performed 31 days post-implant procedure, indicating no vocal cord paresis, aspiration, or asymmetry. Two additional laryngoscopies were performed as the patient continued to experience persistent and occasionally severe hoarseness. These exams confirmed bilateral bowing of the vocal folds, incomplete glottic closure, and a small cyst in the right vocal fold. Moderate dysphonia was observed, characterized by breathy raspiness, strain, and vocal weakness. The patient was advised to increase hydration and pursue voice therapy. The patient reported significant improvement after attending several voice therapies, but mild hoarseness persists.

## Discussion

Current treatment approaches for chronic inflammatory diseases focus on discovering safer and more effective therapies. The promising results of neuroimmune modulation in reducing disease severity in RA have opened a new field for researchers to explore. We describe a novel system and its surgical implantation procedure and safety under investigation in the RESET-RA study in patients with RA. The age and sex demographics of the patients enrolled in this study were representative of patients with RA treated at the referring clinics. The initial safety analysis of this study shows that implant procedures were completed without intraoperative complications, infections, or surgical revisions. In addition, no unanticipated adverse device effects were reported. The two procedure-related SAEs were anticipated events known to be associated with the FDA-approved vagus nerve stimulation implant procedures.

Electrical stimulation of the vagus nerve has been used in the United States since 1997 as an adjunctive treatment for patients with drug-refractory epilepsy (Ben-Menachem et al. 1999; Morris and Mueller 1999;  Handforth et al. 1998; Klinkenberg et al. 2012; The Vagus Nerve Study Group 1995) and difficult-to-treat depression (Rush et al. 2005a, b). Since the first human implant of a vagus nerve stimulation device in 1988 (Penry et al. 1990), more than 125,000 such devices have been implanted in patients worldwide (Fisher et al. 2021; Fetzer et al. 2021). A novel system to administer vagus nerve stimulation therapy to patients with RA features several benefits intended to improve the safety of the implantation procedure over traditional vagus nerve stimulation devices. While the technique used to isolate the nerve is similar, the pulse generator used in the RESET-RA study has a significantly smaller form factor and integrated electrodes that obviate the need to tunnel electrical leads from the target stimulation site in the neck to a separate pulse generator in the chest. Furthermore, the miniaturized, integrated form factor of this study’s device minimizes manipulation of the nerve as the electrodes are not required to be coiled around the nerve, and there are no tethered leads. The longer battery life of at least ten years for the study’s pulse generator is another advantage compared to other traditional implanted vagus nerve stimulation devices, which have batteries that last 3 to 8 years. These improvements over traditional vagus nerve stimulation devices may help alleviate some common adverse events associated with implant procedures, frequent battery replacements, lead fractures, and scar tissue formation while improving compliance and adherence to treatment, a known limitation of current drug-based RA therapies (Waimann et al. 2013).

Electrical stimulation of the vagus nerve is generally well tolerated, and severe side effects are rare. The most common effects are hoarseness, cough, shortness of breath, and paresthesias. These typically appear during stimulation and resolve once stimulation is turned off (Ben-Menachem 2002; O’Reardon et al. 2006). Typical duty cycles are 30 s of stimulation every 5 min. Typical pulse frequencies for treating epilepsy and depression range between 20 and 30 Hz with pulse widths of 250–500 µs (Livanova 2020). Higher pulse frequencies and widths may be associated with reductions in heart rate (Ardell et al. 2017; Heck et al. 2002). Minimizing these concerns, the device in this study for treating RA uses an extremely low duty cycle (60 s per day), a lower pulse frequency and pulse width of 10 Hz and 250 µs, respectively, that were well tolerated, safe, and did not demonstrate an impact on heart rate in several animal studies and previous human clinical studies of RA (Koopman et al. 2016; Genovese et al. 2020; Levine et al. 2018a, b).

Voice alterations, dyspnea, vocal cord palsy, coughing, and neck and throat pain are surgery-related side effects reported in approximately 17% of the patients undergoing implantation of traditional vagus nerve stimulation devices (Milby et al. 2009; Kahlow and Olivecrona 2013). These effects typically occur within days after surgical procedure and are relatively easier to manage compared to typical adverse effects, such as serious infections, malignancies, and major cardiac events, associated with biological and targeted synthetic DMARD therapy, which is currently the mainstay of RA treatment (Frisell et al. 2023; US FDA 2021).

In addition to the case reported here, another single case of left vocal cord paresis was reported in this novel device’s first human RA study that occurred within 24 h after the procedure. Vocal cord paresis has been proposed to likely occur due to acute injury to the vagus nerve or recurrent laryngeal nerve (Aalbers et al. 2015). In most cases, the vagus nerve is the site of injury, as the recurrent laryngeal nerve is not knowingly disturbed. These complications may be related to branching of the vagus nerve, especially the superior and the (non-) recurrent inferior laryngeal nerve. Left is the most common side of cervical vagus nerve stimulation device implantation, where branching may be as high as 12% (Hammer et al. 2015). In a review of 51,882 epilepsy vagus nerve stimulation device implantations, complications of vocal cord paresis occurred significantly more frequently with coil size of 2 mm versus 3 mm because of differences in rigidity as a function of the radius of curvature of the leads and subsequent difficulty in attaching smaller stiffer coils that ultimately led to differences in the risk of vagus nerve injury (Panebianco et al. 2016). The leadless integrated electrodes featured on the novel pulse generator used in this study sit in close approximation to the vagus nerve but do not wrap around it, which in part may alleviate mechanical stress on the nerve.

## Conclusions

Despite the success of currently available drugs for treating RA, many patients do not achieve long-term responses to these therapies (Radawski et al. 2019; Winthrop et al. 2020). New therapeutic approaches employing different mechanisms of action, such as activating the inflammatory reflex, are needed. Data from this study demonstrate that neuroimmune modulation using a novel miniaturized device was safe, and the surgical procedure and device were well tolerated. Utilization of neuroimmune modulation for the treatment of RA may provide a safer, more compliant, and cost-effective treatment compared to biologic DMARD therapy. When fully completed, the clinical efficacy results of this study will provide more definitive conclusions about the potential of neuroimmune modulation to reduce the severity of chronic inflammatory diseases such as RA.

### Supplementary Information

**Additional file 1: Supplemental Table 1.** Inclusion and exclusion criteria. **Supplemental Table 2.** Prohibited medical and surgical interventions.

## Data Availability

All data generated or analyzed during this study are included in this published article and its supplementary information files.

## Abbreviations

ACPA: Anti-citrullinated protein antibody
ACR: American college of rheumatology
AE: Adverse event
b/ts DMARD: Biologic or targeted synthetic disease-modifying antirheumatic drug
CDAI: Clinical disease activity index
CTLA-Ig: Cytotoxic T lymphocyte associated antigen-4 immunoglobulin
DAS28(CRP): Disease activity score in 28 joints with C-reactive protein
HAQ-DI: Health assessment questionnaire disability index
hsCRP: High sensitivity C-reactive protein
ITT: Intent to treat
JAKi: Janus kinase inhibitor
RA: Rheumatoid arthritis
RF: Rheumatoid factor
SD: Standard deviation
SJC28: Swollen joint count of 28 joints
TJC28: Tender joint count of 28 joints
TNF: Tumor necrosis factor
