# Supplementary material for: Clinical safety and feasibility of a novel implantable neuroimmune modulation device for the treatment of rheumatoid arthritis: initial results from the randomized, double-blind, sham-controlled RESET-RA study
Source: Bioelectron Med. 2024 Mar 13;10:8. doi: 10.1186/s42234-023-00138-x (PMC10935935; doi:10.1186/s42234-023-00138-x)
Supplement: Supplementary file 1 — Additional file 1: Supplemental Table 1. Inclusion and exclusion criteria. Supplemental Table 2. Prohibited medical and surgical interventions. [file 42234_2023_138_MOESM1_ESM.docx]

**Additional file 1**

**Supplemental Table 1****. Inclusion and Exclusion Criteria**

| Key inclusion criteria | Key exclusion criteria |
| --- | --- |
| Male or female and 22-75 years of age, inclusive | Received a biologic or targeted synthetic DMARD within the defined number of days prior to implant procedure:  Biologic DMARD: abatacept weekly SC (14 days) or monthly IV (30 days), adalimumab (21 days), anakinra (2 days), certolizumab every 2 week (21 days) or every 4 weeks (30 days), etanercept (14 days), golimumab SW every 4 weeks (30 days) or every 8 weeks IV (60 Days), infliximab every 4 weeks SQ (30 days) or every 6 weeks SC (40 days) or every 8 weeks SC (60 days), rituximab (120 days), sarilumab (28 days), or tocilizumab IV (30 days) or SC (21 days)  Targeted synthetic DMARD: baricitinib (2 days), tofacitinib (2 days), upadacitinib (2 days)  Prior JAKi user not within the protocol limit of 10% of the study stage 1 population (n=6)  Received intra-articular corticosteroid injection within 30 days of implant procedure  Received glucocorticoids at doses greater than 10 mg QD of prednisone (or equivalent) or have been receiving an unstable dosing regimen within 14 days of informed consent  Have started treatment with NSAIDs or have been receiving an unstable dosing regimen of NSAIDs within 14 days of informed consent. Over-the-counter use of NSAIDs is permissible |
| Provided written informed consent | Current, regular use of nicotine products, including but not limited to cigarettes or other nicotine-containing products such as cigars, pipe tobacco, chewing tobacco, patches, gums, lozenges, or electronic cigarettes. Subject does not agree to abstain from using nicotine containing products throughout study participation. |
| Have active, moderate-to-severe RA, defined as at least 4/28 tender joints and 4/28 swollen joints | Woman who is either pregnant or breast feeding  Woman of childbearing potential who does not agree to use an effective method of contraception throughout the study |
| Demonstrated an inadequate response, loss of response, or intolerance to at least 1 approved for RA biologic or targeted synthetic DMARD, including JAKi (limited to 10% of the stage 1 study population) | Untreated or poorly controlled psychiatric illness or history of substance abuse |
| Receiving treatment with at least 1 conventional synthetic DMARD for at least 12 weeks prior and on a continuous, non-changing dose for at least 4 weeks prior to screening and able to continue the same stable dose through Week 12. A stable dose equivalent to ≥ 10 mg/week of MTX is acceptable. Combinations of oral DMARDS such as MTX and hydroxychloroquine or sulfasalazine are acceptable. | Active infection requiring treatment with antibiotics or corticosteroids at screening |
| Willing and able to comply with protocol requirements | Significant immunodeficiency due to underlying illness (e.g., active, untreated HBV/HCV and HIV positive) |
|  | History of CVA or transient ischemic attack, or diagnosis of cerebrovascular fibromuscular dysplasia |
|  | History of clinically significant cardiovascular disease, including cardiomyopathy with ejection fraction < 40%, myocardial infarction, unstable angina, or diagnosis of congestive heart failure (NYHA Class III or IV) in the preceding 12 months. Subjects with a history of cardiovascular disease must obtain clearance from a cardiologist |
|  | Neurological syndromes including multiple sclerosis, Alzheimer’s disease, or Parkinson’s disease |
|  | Active fibromyalgia despite ongoing treatment that in the opinion of investigator is confounding the tender and swollen joint count |
|  | Known clinically significant cerebrovascular atherosclerotic disease including contralateral carotid artery disease (non-implanted side) lung disease causing clinically significant dyspnea at screening |
|  | History of or pre-surgical X-ray that shows cervical spine disorder or instability that would preclude safe endotracheal anesthesia |
|  | Calculated creatinine clearance less than or equal to 50 mL/min/1.73 m2 at Screening |
|  | History of left or right carotid surgery (e.g., carotid endarterectomy or stent); unilateral or bilateral vagotomy; partial or complete splenectomy |
|  | Recurrent vasovagal syncope episodes |
|  | Clinically significant cardiac rhythm disturbances, or ECG findings of atrioventricular block exceeding first degree, or cardiac conduction pathway abnormalities other than isolated right bundle branch block or isolated left anterior fascicle block on Screening ECG |
|  | Cancer within last 3 years, except fully resected basal or squamous skin cancer, fully resected cervical carcinoma in situ, or fully treated mammary ductal carcinoma in situ that has been in clinical remission for at least 3 years |
|  | Clinically significant esophageal dysfunction such as dysphagia, odynophagia, or esophagitis |
|  | Pre-existing clinically significant vocal cord dysfunction or hoarseness, or history of vocal cord polyps |
|  | Active and uncontrolled peptic ulcer disease |
|  | Implanted active medical devices (e.g., cardiac pacemakers, automatic implantable cardioverter defibrillators, drug pumps), or likely need for implantation of such devices within 6 months |
|  | Uncontrolled asthma, chronic obstructive pulmonary disease, or any other pulmonary disease such as interstitial lung disease causing clinically significant dyspnea |
|  | Limited life expectancy due to terminal disease |
|  | Hypersensitivity/allergy to MRI contrast agents and/or unable to perform MRI (e.g., claustrophobia) |
|  | Unable to use or wear Energizer |
|  | Requires treatment by any of the prohibited interventions (see Table 2) |
|  | Currently participating in another clinical research study with an investigational drug or  medical device |
|  | Have taken an investigational drug for RA within the defined time period prior to Implant Procedure:  a. Have taken an investigational drug consisting of biologic agents within 30 days prior to Implant Procedure  b. Have taken an investigational drug consisting of small molecules within 30 days or 5 times the pharmacokinetic half-life, whichever is longer, of Implant Procedure |
|  | Uncontrolled hypertension, diabetes mellitus |
|  | Any comorbidity or current status of subject’s physiological fitness that in the surgeon’s or anesthesiologist’s opinion represents safety concerns that make the subject medically unfit for the implant procedure or may prevent proper placement of MicroRegulator and POD |
|  | A positive COVID-19 test between informed consent and Implant Procedure |
|  | Works or resides in an environment where radio frequency (RF) exposure is higher than levels deemed safe for normal daily activity by the general public (e.g., near radio or television broadcasting antennas, radar systems, industrial heaters and sealers) |
|  | Any condition that, in the investigator’s opinion, may preclude completion of follow-up assessments during the 12-week study duration (e.g., a medical condition that may increase the risk associated with study participation or may interfere with interpretation of study results, inability to adhere to the visit schedule, or poor compliance with treatment regimen) |

## **Supplemental Table 2. Prohibited Medical and Surgical Interventions**

| The following medications and medical treatments are prohibited during study participation: |
| --- |
| 1. Steroid pulse therapy through Week 12 |
| 1. Biologic or targeted synthetic DMARDs through Week 12 |
| 1. Intra-articular corticosteroid injection through Week 12 |
| 1. Ionic or nonionic linear chelated gadolinium-based contrast agents for MRI studies such as gadodiamide, gadoversematide, gadofosvest trisodium, gadoxetate disodium, gadopentate dimeglumine, and gadobenate dimeglumine |
| 1. External electrical stimulation devices (e.g., TENS units) on the neck |
| 1. Electrically active implantable medical devices (e.g., cardiac pacemakers, automatic implantable cardioverter-defibrillators) |
| 1. Extra-corporeal shock wave lithotripsy in the cervical spine region |
| 1. Short-wave diathermy, microwave diathermy, or therapeutic ultrasound diathermy. Diagnostic ultrasound is not included in this contraindication |
| 1. Electrosurgery (electrocautery or radio frequency ablation devices) in the cervical spine region |
| 1. Monopolar electrocautery |
| 1. Any investigational therapy |
| 1. Therapeutic radiation |
